# Comparable endocrine and neuromuscular adaptations to variable vs. constant gravity-dependent resistance training among young women

**DOI:** 10.1186/s12967-020-02411-y

**Published:** 2020-06-15

**Authors:** Hamid Arazi, Lida Salek, Elham Nikfal, Mani Izadi, James J. Tufano, Bradley T. Elliott, Matt Brughelli

**Affiliations:** 1 Department of Exercise Physiology, Faculty of Sport Sciences, University of Guilan, 10 th km of Tehran Road- Khalij-e-Fars Highway, Zip Code: 4199843653 Rasht, Iran; 2 Department of Physical Education, Bandar-e-Anzali Branch, Islamic Azad University, Bandar-e-Anzali, Iran; 3 Faculty of Physical Education and Sport, Charles University, Prague, Czech Republic; 4 Translational Physiology Research Group, School of Life Sciences, University of Westminster, London, UK; 5 Sports Performance Research Institute New Zealand (SPRINZ), AUT Millennium, Auckland University of Technology, Auckland, New Zealand

**Keywords:** Chain-loaded resistance training, Traditional resistance training, Hormone, Muscle, Strength

## Abstract

**Background:**

Variable resistance has been shown to induce greater total work and muscle activation when compared to constant resistance. However, little is known regarding the effects of chronic exposure to variable resistance training in comparison with constant resistance training. The aim of the present study was therefore to examine the effects of chain-loaded variable and constant gravity-dependent resistance training on resting hormonal and neuromuscular adaptations.

**Methods:**

Young women were randomly assigned to variable resistance training (VRT; n = 12; age, 23.75 ± 3.64 years; and BMI, 26.80 ± 4.21 kg m^−2^), constant resistance training (CRT; n = 12; age, 23.58 ± 3.84 years; BMI, 25.25 ± 3.84 kg m^−2^), or control (Con; n = 12; age, 23.50 ± 2.93 years; BMI, 27.12 ± 12 kg m^−2^) groups. CRT performed 8-week total-body free-weight training three times per week with moderate-to-high intensity (65–80% 1RM; periodized). VRT was the same as CRT but included variable resistance via chains (15% of total load). Resting serum samples were taken before and after the 8-week intervention for GH, IGF-1, cortisol, myostatin, and follistatin analyses.

**Results:**

Both VRT and CRT groups displayed moderate-to-large significant increases in GH (197.1%; ES = 0.78 vs. 229.9%; ES = 1.55), IGF-1 (82.3%; ES = 1.87 vs. 66%; ES = 1.66), and follistatin (58.8%; ES = 0.80 vs. 49.15%; ES = 0.80) and decreases in cortisol (− 19.9%; ES = − 1.34 vs. − 17.1%; ES = − 1.05) and myostatin (− 26.9%; ES = − 0.78 vs. − 23.2%; ES = − 0.82). Also, VRT and CRT resulted in large significant increases in bench press (30.54%; ES = 1.45 vs. 25.08%; ES = 1.12) and squat (30.63%; ES = 1.28 vs. 24.81%; ES = 1.21) strength, with no differences between groups.

**Conclusions:**

Implementing chain-loaded VRT into a periodized resistance training program can be an effective alternative to constant loading during free-weight RT among untrained young women.

## Background

Resistance training (RT) induces acute mechanical and physiological responses that play a role in chronic adaptations including muscular strength, hypertrophy, and performance. Although other factors are at play, the acute responses to RT and subsequent tissue remodeling are primarily mediated via the neuroendocrine system [1]. More specifically, some hormones and cytokines have received further attention in studies on the responses and adaptations to RT programs due to their pivotal action in muscle anabolism. For example, growth hormone (GH) and insulin-like growth factor-1 (IGF-1) are some of the most studied, having a major role in promoting tissue anabolism [2]. On the other hand, cortisol and myostatin impose suppressive effects on muscle growth mainly thorough protein degradation in muscle cells and inhibitory roles on the expression of myogenic regulatory factors, respectively [2, 3]. Besides, follistatin is a putative inhibitor of myostatin, which can contribute to elevated anabolic responses [3].

Interestingly, regardless of the exact signaling mechanisms, the total amount of work performed and greater muscle volume activated during RT are of the primary mediators for stimulating the anabolic responses, endocrine adaptations, and subsequent skeletal muscle adaptations including muscle hypertrophy [4]. Hence, since the contractile function of skeletal muscle is largely dependent on muscle size (together with fiber type) [5], maximizing the amount of external work during RT may help further stimulate the endocrine system and skeletal muscle adaptations, thereby improving neuromuscular performance.

During traditional, gravity-dependent RT, a constant resistance is most often used, executing lifts with free-weights or performing exercises with pin-loaded machines. It has been suggested that constant resistance cannot fully stimulate the neuromuscular system during exercises with an ascending strength curve (such as squat and bench press) where the amount of force produced by the involved muscle group(s) increases during the latter stages of concentric muscle actions (the type of muscle action that occurs when the length of the muscle shortens as tension is produced). Considering the importance of total work (i.e. repetitions × sets × average concentric force) for stimulating neuromuscular adaptations [6], the use of lower percentages of maximal force during the latter phases (compared to initial phases) of concentric muscle actions may not encourage optimal adaptations. In other words, during this type of exercises in traditional RT, load remains constant, while the amount of force can be produced by muscle(s) involved increases as the movement progresses in concentric phase. Then, the magnitude of mechanical stimulus is not the same across the range of motion.

On the other hand, variable resistance methods involve a variation in the magnitude of the resistance throughout the exercise’s range of motion. An advantage of variable resistance methods includes the possibility to match the increases and decreases in strength (strength curve) throughout an exercise’s range of motion [7]. This could result in exerting near-maximal force by the muscle(s) involved throughout the range of motion. Accordingly, the main purpose of chain-based variable RT (VRT) is to progressively increase the resistance as movement progresses in the concentric range of motion, providing an ascending strength curve-matched load [8].

Previous research suggests that variable resistance can result in greater total work [6], and higher muscle activity in the eccentric phase (the type of muscle action that occurs when the length of the muscle increases as tension is produced) [9] and final portions of the concentric phase [10] of a movement compared to constant RT (CRT) and can potentially activate the neuromuscular system to enhance subsequent lifting performance [11]. Although these acute findings are worth considering, research investigating the chronic effects of VRT in untrained individuals is less common, with some researchers finding that variable resistance via elastic bands increased strength greater than CRT [12] while others noted that strength gains were equivocal between variable and constant resistance [13]. In this regard, a recent review [14] concluded that VRT programs resulted in better performance gains compared to constant resistance in a wide range of samples, supporting the conclusions of a recent meta-analysis [15] that also reported greater gains using variable resistance. However, a subgroup analysis by training status indicated that differences between variable and constant resistance were not significant among untrained participants despite effect sizes favoring variable resistance. Nevertheless, women have been largely underrepresented in these studies, highlighting the need for more research in female populations.

Thus, there is limited evidence demonstrating the chronic neuromuscular adaptations of VRT compared to CRT, and to our knowledge, data on resting hormonal adaptations to VRT are lacking. Additionally, considering that the majority of previous research has investigated variable resistance using bands, mostly in trained men, the effects of variable resistance using chains in untrained women is largely unexplored. While, the increase in resistance follows a pattern which is curvilinear for band-loaded and linear for chain-loaded VRT, and these inequalities may result in relatively different adaptations. If variable resistance using chains could increase the force requirements for a given number of repetitions in this population, total work could increase and the hormonal and neuromuscular responses to training may be magnified compared to traditional CRT. Therefore, the purpose of this study was to examine the hormonal and strength adaptations after eight weeks of CRT or VRT in untrained young women.

## Methods

### Study design

**Fig. 1:**
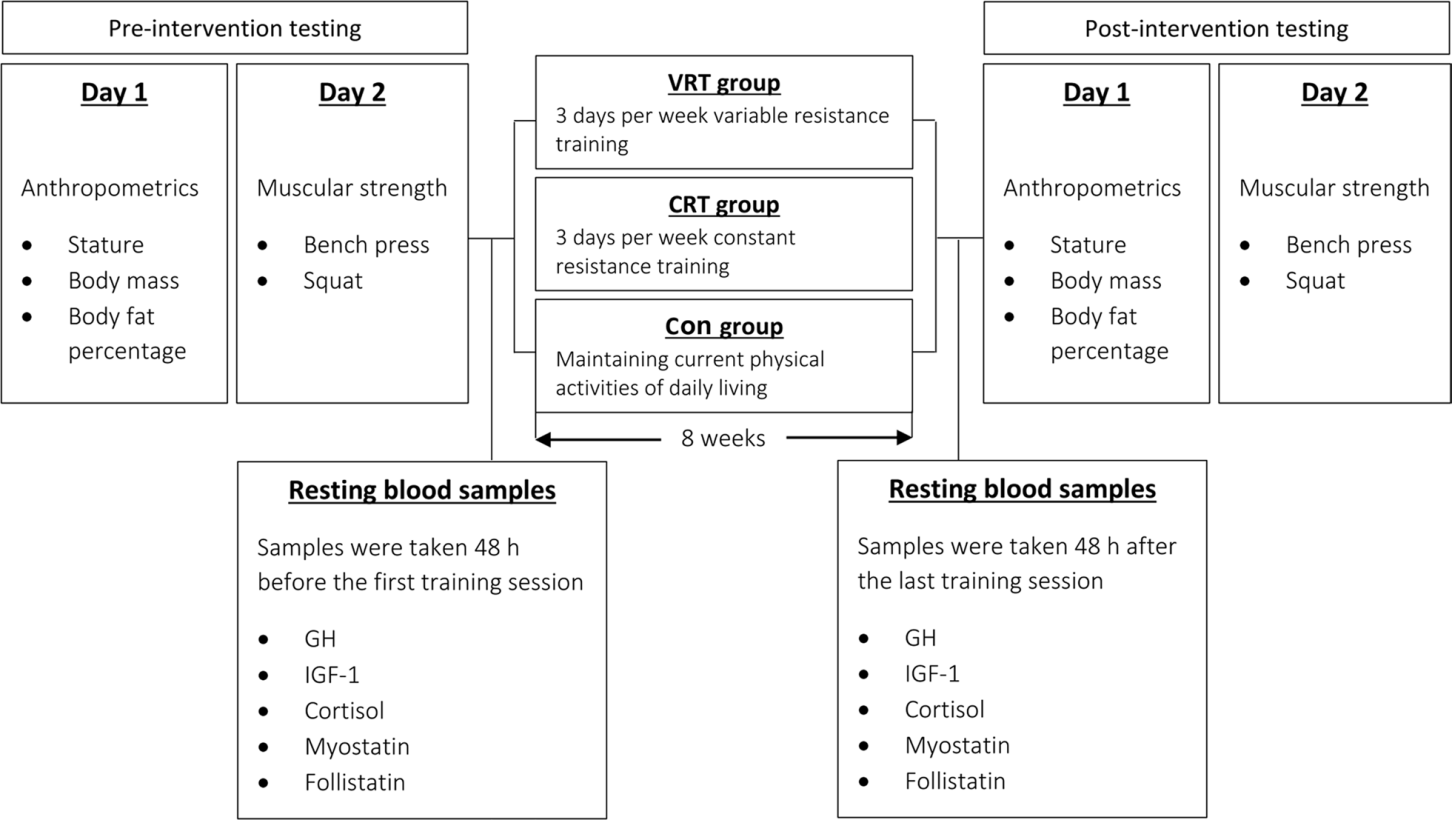
Experimental design

The present study is a randomised controlled intervention design. Anthropometric characteristics, muscular strength, and resting hormonal levels were assessed for all participants prior to and after the intervention program (Fig. 1). Participants were randomly assigned to one of the following three groups: (i) variable RT (VRT; n = 12); (ii) constant RT (CRT; n = 12); and (iii) control (Con; n = 12). All participants were instructed to maintain their current lifestyle physical activity and dietary habits during the period of intervention. Further, those in VRT and CRT groups performed their specified RT programs three times a week for eight consecutive weeks.

### Participants

The procedures were approved by the Institutional Review Committee of the Islamic Azad University, Bandar-e-Anzali Branch, Iran (ref. 13950630-2016) according to the current national laws and regulations and to the latest version of the Helsinki Declaration. G-Power software, version 3.1.9.4 [16], was used to calculate the sample size required for the present study. Using a statistical power of 0.85 and an effect size of 0.75, a total sample size of at least 28 participants was necessary to test our hypothesis at p< 0.05.

Thirty-six young, eumenorrheic women provided written informed consent to participate in the present study. All the procedures and risks were explained before the participants gave their written consent, and they were informed that they could withdraw from the study at any time. A questionnaire regarding medical, orthopedic injury, and RT history was completed. None of them had any experience in systematic RT for ≥ 1 year, but all the participants were familiar with the basics of the RT. Participants had no history of injury during the preceding 6 months or any medical conditions precluding them from participating in high-intensity RT. A summary of participants’ characteristics is provided in Table 1.

**Table 1:** Physical characteristics of study participants

	VRT group (n = 12)	CRT group (n = 12)	Con group (n = 12)	Total (n = 36)
Age (y)	23.75 ± 3.64	23.58 ± 3.84	23.50 ± 2.93	23.61 ± 3.39
Stature (cm)	164.3 ± 8.30	166 ± 5.41	165.4 ± 6.47	165.2 ± 6.67
Body mass (kg)				
Pre	72.62 ± 14.72	69.58 ± 10.59	74.61 ± 16.84	72.27 ± 14.03
Post	73.66 ± 13.39	70.75 ± 9.27	75.16 ± 16.50	73.19 ± 13.13
BMI (kg m^−2^)				
Pre	26.80 ± 4.21	25.25 ± 3.84	27.12 ± 12	26.39 ± 4.37
Post	27.23 ± 4.08	25.68 ± 3.39	27.33 ± 5	26.75 ± 4.16
% Body fat				
Pre	26.41 ± 3.39	27.08 ± 2.96	26.75 ± 3.38	26.75 ± 3.17
Post	23.75 ± 3.86	23.75 ± 2.37	27.16 ± 3.83	24.88 ± 3.70

Values are mean ± SD

### Testing procedures

Assessments before and after the eight weeks of RT programs were completed at the same time of the day, as circadian and/or diurnal rhythms may influence human hormonal secretion and muscular strength [1]. Additionally, measurements were taken by the same experienced examiners on both occasions, who were blinded regarding the group assignments.

### Anthropometric measures

Body stature was measured to the nearest 0.1 cm using a stadiometer (Seca 222, Terre Haute, Ind., USA), body mass was determined via a medical weighing scale (Tanita, BC-418MA, Tokyo, Japan), and body fat percentage was assessed through 3-site measurements of skinfold thickness (triceps, suprailiac, and thigh) using a Slim Guide skinfold caliper (Creative Health Products, Plymouth, MI, USA), with fat free mass (FFM) thus = body mass – (body mass × percentage fat mass). All the procedures were performed by the same examiner on both occasions and in accordance with the guidelines by the International Society for the Advancement of Kinanthropometry [17]. The equations provided by Jackson et al. [18] and Siri [19] were used to evaluate body density and body fat percentage, respectively.

### Muscular strength assessment

The bench press and squat were used to evaluate the upper body and lower body muscular strength, respectively. Although the 1-repetition maximum (1RM) test is widely used and is considered safe when performed correctly, novice untrained participants may be unwilling to continue increasing the load over several trials until reaching the maximal values. Further, the application of maximal loads may expose the novice participants to the risk of injury. Therefore, a sub-maximal test was employed, from which the 1RM was estimated. Participants underwent a 15-min warm-up including 5–10 min of stretching and mobilization exercises focusing on hip, knee, shoulder, and elbow flexors and extensors. For each of the tests, participants did 2–10 repetitions at submaximal loads. Then, up to 3 trials were allowed to reach the lower-repetition, heavier-load end of the spectrum. Between sets, 3 min of rest was allowed to provide sufficient recovery. Finally, the equation provided by Brzycki [20] was used to estimate the maximal strength for each exercise.

### Blood analyses

Pre- and post-intervention resting blood samples were taken 48 h before the first session of RT and 48 h after the last training session, respectively. After 12 h fasting and 8 h sleep, 5 mL of blood was collected from an antecubital vein using sterile techniques, the blood was transferred into serum tubes, and centrifuged for 15 min at 1100*g*. The resulting serum was pipetted into tubes and kept in the freezer (− 70 °C) for later analyses. All of hormone evaluations were made in duplicate. Therefore, enzyme-linked immunosorbent assays were used to analyze GH, IGF-1, cortisol (DiaMetra kit, Milano, Italy), myostatin, and follistatin (Shanghai Crystal Day Biotech kits, Wuhan, PRC). The intra-assay variance was set based on a coefficient of variation of < 6%.

### Training program

Participants in VRT and CRT groups attended three training sessions per week on Mondays, Wednesdays, and Saturdays from about 4 to 6 p.m. with an average duration of 45 to 75 min (depending on the phase of training periodization) per session. The RT program utilized a linear periodization strategy. Since previous research has shown that peak force is significantly increased when combining variable resistance and free weights at heavier loads compared to lighter loads [21], the present training program included loads of 65% 1RM and over. Participants in the CRT group performed bench press and squat exercises using free weights, but participants in the VRT group performed the same exercises with the inclusion of chains to free weights.

Instructions for the squat and bench press exercises were as follows. For the squat exercise, the participant maintained an upright position, and the bar was grasped with both hands and positioned on the shoulder behind the neck and feet were placed shoulder width apart. Then, the participant descended until the posterior part of the thigh was aligned parallel to the floor and regained the start position thorough ascending. For the bench press exercise, participants maintained a supine position on the bench with the head and trunk supported by the bench, the knees were bent and the feet on the floor. The bar was grasped with both hands with a distance greater than the shoulder width and less than 81 cm (the greatest width allowed by International Powerlifting Federation) between the palms, then was lowered to touch the chest and was lifted upward until elbows reached full extension. The range of motion and proper technique of exercise were ensured by the same spotter (a research staff member). Since the participants were untrained, no any exact exercise tempo was set. However, tempos were mostly around 2–2 (2-s concentric and 2-s eccentric actions).

The training load for each exercise during VRT was reduced by 15% and replaced chains (¼-inch chains with a total length of 150 cm) that were attached to each end of the barbell through a spring clip. Additionally, the VRT and CRT groups performed lateral pull downs and lying leg curls using machines (Inpars Co, Isfahan, Iran) to maintain a balance of training stimulus between agonist and antagonist muscles on both upper body and lower body. During these machine-based exercises, chains were not used. Compliance to all VRT and CRT sessions was monitored by the investigators, and in the few cases that a participant did not attend a session, an alternative session was allocated within the same week. A summary of resistance training program can be found in Table 2.

**Table 2:** Resistance Training Program

Week	Sets	Reps	Intensity (% 1RM)	Rest (s)
1	3	10	65	120
2	3	10	65	120
3	3	10	70	120-180
4	3	12	70	120-180
5	3	12-10-10	75	120-180
6	4	12-10-10-8	75	120-180
7	4	10-10-8-8	80	180
8	5	10-10-8-8-6	80	180

Exercises were the same for the 8-week resistance training program including bench press, squat, leg curl, and lateral pull down

### Statistical analyses

All data were analyzed using SPSS statistical software version 26 (SPSS, Inc., Chicago, IL, USA). Normality of data and homogeneity of variances were checked using Shapiro–Wilk and Levene’s tests, respectively. Values are presented as means ± standard deviations. Percentage changes were calculated as ([post training − baseline]/baseline × 100). The analyses of covariance and post hoc tests with Bonferroni adjustments were conducted to examine the differences in pre- to post-intervention changes between the 3 groups. The baseline values were covariates, and the post-intervention values were the dependent variables. Then, intra-group differences between pre- and post-intervention values were compared using the paired samples *t* test. The effect sizes (Cohen’s d) plus 95% confidence interval were calculated as in previous studies [22, 23]. The classification for magnitudes of the effect sizes were as follows: ≤ 0.20, trivial; 0.21–0.50, small; 0.51–0.80, moderate; > 0.80, large [24]. Pearson correlation coefficient was used to evaluate the relationship between FFM and hormonal measures. Statistical significance was set at p ≤ 0.05.

## Results

### Hormonal measures

**Fig. 2:**
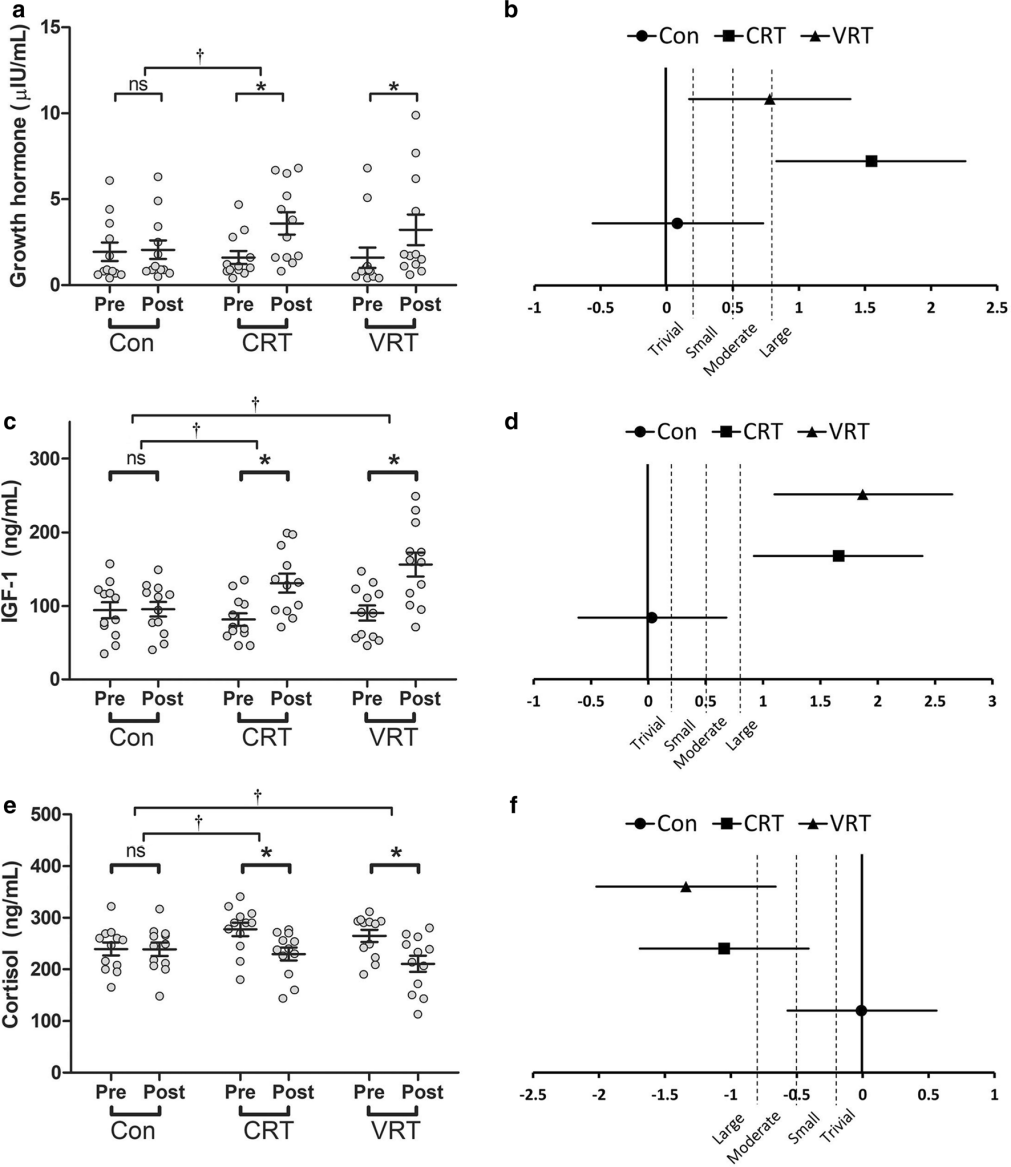
**a** GH pre- to post-intervention values; **b** GH effect sizes; **c** IGF-1 pre- to post-intervention values; **d** IGF-1 effect sizes; **e** cortisol pre- to post-intervention values; **f** cortisol effect sizes. For within-group comparisons: ^ns^ not significant compared to baseline, p > 0.05; *Significantly different from baseline, p ≤ 0.05. For between-group comparisons: ^†^Significantly different compared to the Control group, p ≤ 0.05

After the 8-week training period, there were moderate and large significant increases in GH levels for the VRT (*p* = 0.014; ES = 0.78, 95% CI = 0.17 to 1.39; Δ of 197.1 ± 320.4%) and CRT (*p* = 0.003; ES = 1.55, 95% CI = 0.83 to 2.26; Δ of 229.9 ± 440.4%) groups, respectively (Fig. 2a, b). However, no significant difference was found between the VRT and CRT groups (*p *>0.05) (Fig. 2a).

Similarly, large significant increases in IGF-1 were observed for the VRT (*p* = 0.001; ES = 1.87, 95% CI = 1.10 to 2.65; Δ of 82.3 ± 57.6%) and CRT (*p* = 0.001; ES = 1.66, 95% CI = 0.92 to 2.39; Δ of 66 ± 44.1%) groups. Further, both the VRT and CRT demonstrated significantly greater increases in IGF-1 than the Con group (*p* = 0.004 and *p* = 0.001, respectively) (Fig. 2c, d). However, no significant difference was found between the VRT and CRT groups (*p *>0.05) (Fig. 2c).

Both the VRT and CRT groups also exhibited significantly greater decreases in resting cortisol compared to the Con group (*p* = 0.004 and *p* = 0.024, respectively). Nevertheless, 2 training groups showed no significant differences (*p *>0.05) in the magnitude of changes (Fig. 2e). Plus, the decreases in cortisol levels were large and significant for the VRT (*p* = 0.003; ES = − 1.34, 95% CI = − 2.02 to − 0.66; Δ of − 19.9 ± 21%) and CRT (*p* = 0.001; ES = − 1.05, 95% CI = − 1.69 to − 0.41; Δ of − 17.1 ± 8.9%) groups (Fig. 2e, f).

**Fig. 3:**
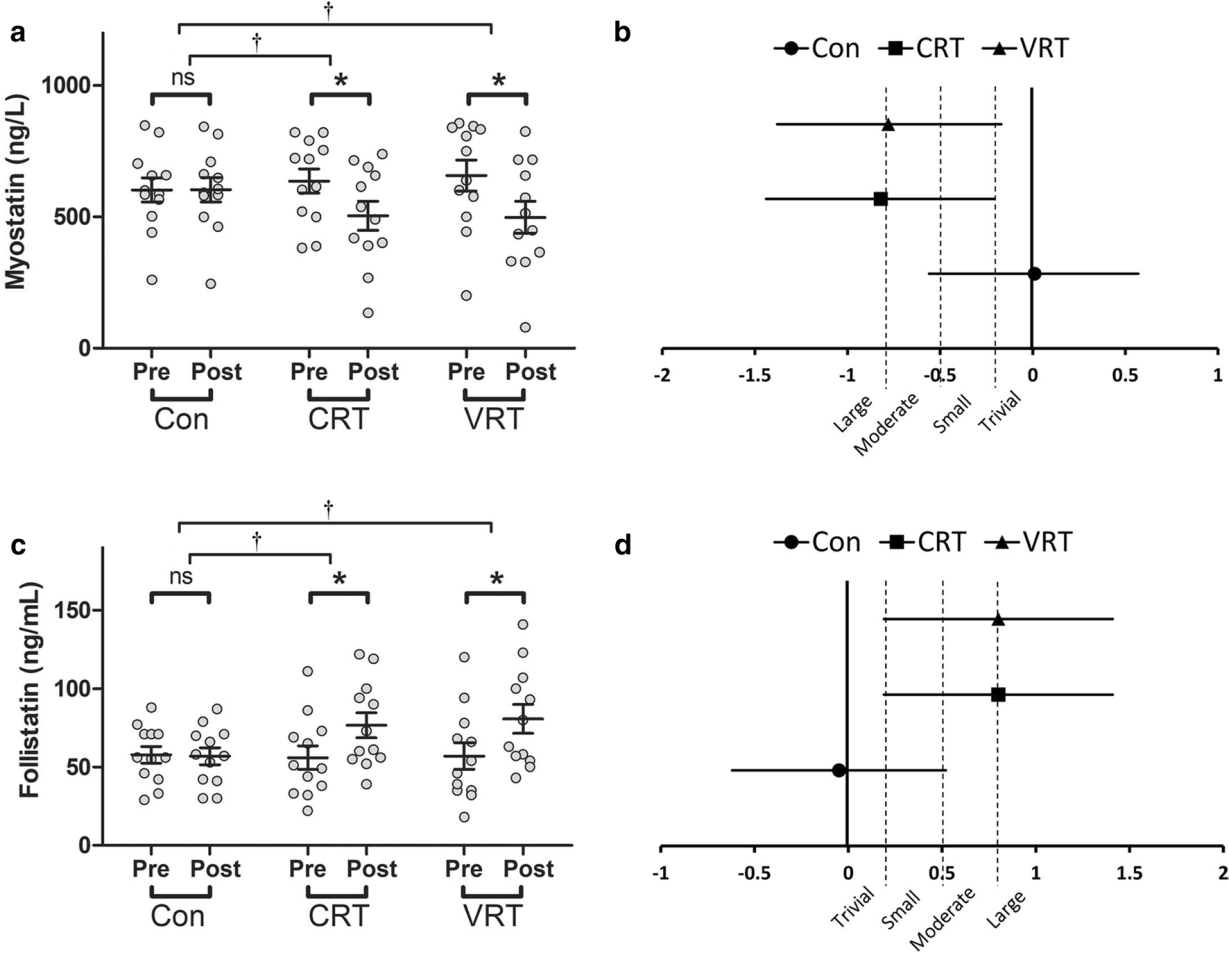
**a** myostatin pre- to post-intervention values; **b** myostatin effect sizes; **c** follistatin pre- to post-intervention values; **d** follistatin effect sizes. For within-group comparisons: ^ns^ not significant compared to baseline, p > 0.05; * Significantly different from baseline, p ≤ 0.05. For between-group comparisons: ^†^Significantly different compared to the Control group, p ≤ 0.05

Moderate and large significant decreases in myostatin were noted for the VRT (*p* = 0.001; ES = − 0.78, 95% CI = − 1.38 to − 0.17; Δ of − 26.9 ± 17%) and CRT (*p* = 0.001; ES = − 0.82, 95% CI = − 1.44 to − 0.21; Δ of − 23.2 ± 15.3%) groups, respectively (Fig. 3a, b). Further, both the VRT and CRT demonstrated significantly greater changes in myostatin compared with the Con group (*p* = 0.001). However, no significant difference was found between the VRT and CRT groups (*p *>0.05) (Fig. 3a).

There were moderate significant increases in follistatin concentrations for the VRT (*p* = 0.001; ES = 0.80, 95% CI = 0.19 to 1.41; Δ of 58.8 ± 66.1%) and CRT (*p* = 0.002; ES = 0.80, 95% CI = 0.19 to 1.41; Δ of 49.15 ± 54.2%) groups (Fig. 3c, d). Although no significant difference was found between the VRT and CRT groups (*p *>0.05), both the VRT and CRT displayed significantly greater changes in follistatin compared with the Con group (*p* = 0.001 and *p* = 0.005, respectively) (Fig. 3c).

### Strength measures

**Fig. 4:**
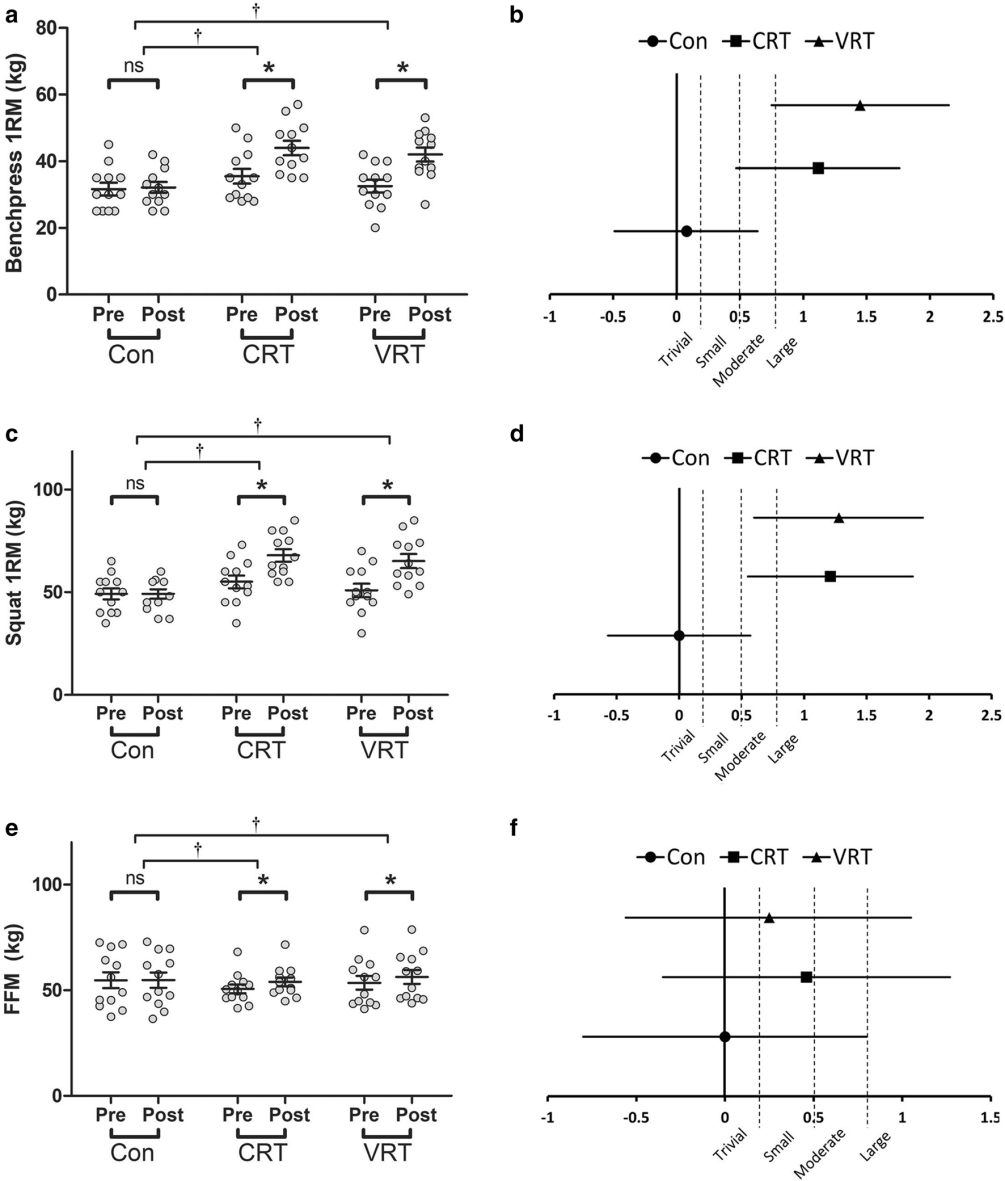
**a** Bench press pre- to post-intervention values; **b** Bench press effect sizes; **c** Squat pre- to post-intervention values; **d** Squat effect sizes; **e** FFM pre- to post-intervention values; **f** FFM effect sizes. For within-group comparisons: ^ns^ not significant compared to baseline, p > 0.05; * Significantly different from baseline, p ≤ 0.05. For between-group comparisons: ^†^Significantly different compared to the Control group, p ≤ 0.05

The Con group exhibited a trivial nonsignificant (p > 0.05) increase in bench press strength after the completion of the 8-week training period. Conversely, there were large significant gains for the VRT (*p* = 0.001; ES = 1.45, 95% CI = 0.75 to 2.15; Δ of 30.54 ± 18.05%) and CRT (*p* = 0.001; ES = 1.12, 95% CI = 0.47 to 1.76; Δ of 25.08 ± 9.87%) groups (Fig. 4a, b). Additionally, the VRT and CRT groups both demonstrated significantly larger gains in bench press strength than the Con group (*p* = 0.001). However, no significant difference was found between the VRT and CRT groups (*p *>0.05) (Fig. 4a).

Further, the Con group did not significantly (p > 0.05) improve the squat strength after the 8-week training program. Conversely, large significant gains were noted for the VRT (*p* = 0.001; ES = 1.28, 95% CI = 0.6 to 1.95; Δ of 30.63 ± 23.13%) and CRT (*p* = 0.001; ES = 1.21, 95% CI = 0.55 to 1.87; Δ of 24.81 ± 10.92%) groups (Fig. 4c, d). Moreover, the VRT and CRT groups both demonstrated significantly larger gains in squat strength than the Con group (*p* = 0.001). However, no significant difference was found between the VRT and CRT groups (*p *>0.05) (Fig. 4c).

### Fat free mass

There were small significant increases in FFM for the VRT (*p* = 0.001; ES = 0.25, 95% CI = − 0.56 to 1.05; Δ of 5.41 ± 2.01%) and CRT (*p* = 0.001; ES = 0.46, 95% CI = − 0.35 to 1.27; Δ of 6.65 ± 3.39%) groups (Fig. 4e, f). Moreover, the VRT and CRT groups both demonstrated significantly larger gains in FFM than the Con group (*p* = 0.001). However, no significant difference was found between the VRT and CRT groups (*p *>0.05) (Fig. 4e).

**Fig. 5:**
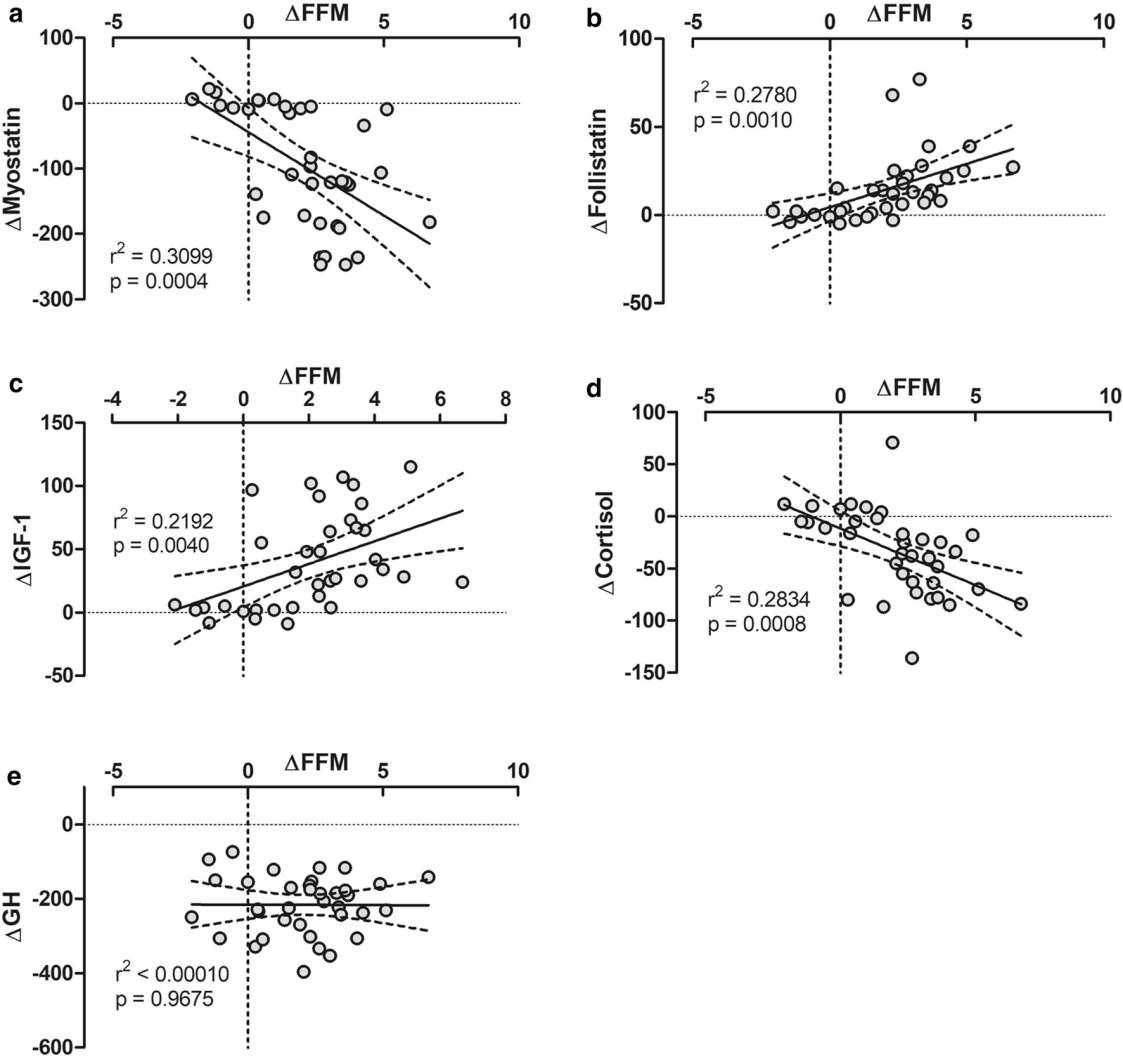
Correlation of FFM with **a** myostatin; **b** follistatin; **c** IGF-1; **d** cortisol; and **e** GH. Delta values are pre- to post-intervention changes

A strong significant negative correlation was noted between the change in FFM and the change in myostatin (r^2^ = 0.0310, p < 0.001) and cortisol (r^2^ = 0.283, p < 0.001) following RT (Fig. 5a, d). Conversely, the change in FFM showed a strong significant positive correlation with the change in follistatin (r^2^ = 0.278, p = 0.001) and IGF-1 (r^2^ = 0.219, p = 0.004) (Fig. 5b, c).

## Discussion

The primary finding of the present investigation was that VRT and CRT both resulted in similar changes in resting hormonal concentrations after the 8-week RT program. With the exception of GH, the VRT group generally demonstrated larger percentages of pre- to post-intervention changes and effect sizes (together with lower and upper bound confidence intervals toward more optimal adaptations) than those observed for CRT, albeit non-significantly different, which is in line with the results of a recent meta-analysis [15].

Greater RT volume and total work, particularly with moderate to high loads and short rest intervals, is generally associated with increased GH responses [4, 25]. However, the results on the chronic adaptations of GH to RT are equivocal. In the present study, both training groups demonstrated a significant increase in resting GH after the training period (Fig. 2) which contradicts the findings of the study by Marx et al. [26] on untrained women. They found no significant change in resting GH values in any of the groups trained using CRT. Additionally, we found no significant correlation between changes in FFM and GH. These contradictions could partially be attributed to pulsatile secretion patterns of GH from anterior pituitary. Frequent samplings are then required to have more exact measures of GH release [27].

Despite no differences between VRT and CRT, VRT may have had a larger effect on IGF-1 compared to CRT (Δ of 82.3% vs. 66%; ES of 1.87 vs. 1.66, and 95% CI of 1.10 to 2.65 vs. 0.92 to 2.39, respectively; Fig. 2). Our findings are consistent with the results of the study by Marx et al. [26] indicating that RT caused significant increase in resting IGF-1 in untrained young women. They found significantly greater elevation in IGF-1 values for women trained using high volume multiple sets compared to their peers in low volume single-set RT. Although no other study exists on adaptations of IGF-1 to VRT compared with CRT, their results support this idea that higher volumes and total work could reinforce the elevation of resting IGF-1 as seen in the present study but to a lesser extent. Besides, we found a greater inter-individual variability in GH and IGF-1 adaptations to VRT compared with CRT. Little is known regarding the heterogeneity of hormonal responses to resistance training. However, it has been shown that genetic contribution influences the individuals’ ability to physiologically adapt to specific physical exercise and exercise mode [28], and there is a link between genetic variations and skeletal muscle characteristics, such as muscle fiber composition which might affect adaptations to training [29].

Similar to IGF-1, resting cortisol levels were not different between VRT and CRT, but VRT may have had a larger effect on decreasing resting cortisol compared to CRT (Δ of − 19.9% vs. − 17.1%; ES of − 1.34 vs. − 1.05, and 95% CI of − 2.02 to − 0.66 vs. − 1.69 to − 0.41, respectively; Fig. 2). Marang [30] investigated the impacts of elastic-loaded VRT on hormonal adaptations of resistance trained men. This study also found no statistically significant differences in cortisol between VRT and CRT groups. While chronic elevations of cortisol can have negative impacts on skeletal muscle [2, 31], an overall reduction of cortisol, as seen in the present study, may indicate a general reduction of protein degradation over time [32], which can be considered as a positive adaptation.

Along the lines of suppressing catabolism and promoting anabolism, myostatin, a known myogenic inhibitor, decreased similarly in VRT and CRT in the present study (Δ of − 26.9% vs. − 23.2%; ES = − 0.78 vs. − 0.82, and 95% CI of − 1.38 to − 0.17 vs. − 1.44 to − 0.2, respectively; Fig. 3). These data support the findings of other studies using moderate to heavy RT [33–36]. Since the expression of myostatin is also mediated by glucocorticoid receptors to induce muscle proteoylsis and the enhancers of the regulatory region within the myostatin gene are responsive to glucocorticoids [37], myostatin may also be downregulated in response to a decline in glucocorticoids such as cortisol and our study supports such a concurrent reduction.

Like myostatin, follistatin is a member of the TGF-β super family and antagonizes myostatin’s action and nullifies its inhibitory role through binding to the ACTIIB myostatin receptor site [38]. Thus, follistatin-mediated action allows greater muscle hypertrophy. Along these lines, follistatin increased similarly (ES of 0.80 in both groups) by 58% and 49% in VRT and CRT, respectively (Fig. 3). The results of the present study confirm those by Attarzadeh Hosseini et al. [39] using high intensity RT on young women. The authors concluded that high intensity provides better anabolic stimulus compared to low intensity RT among young women. Overall, as can be seen in our study and those by others [39], it appears that the intensity of RT plays a major role for inducing significant changes in 2 of the most important factors affecting myogenesis, myostatin and follistatin. In other words, the precise mode of RT may have lesser impact on the changes of these myokines relative to total work completed. Besides, there has been a high variability for myostatin and follistatin concentrations especially in post-intervention values for VRT versus CRT. Possibly, like heterogeneities we found in GH and IGF-1 concentrations, there would be inter-individual differences both at baseline and post-training values. Nevertheless, these differences still remain unknown and further research need to be conducted to unravel this issue. On the other hand, greater variability in post-VRT values compared to CRT, mostly toward optimal values, can partially be influenced by the variability in the baseline values of myostatin and follistatin, as we found further variability in baseline myostatin and follistatin levels for VRT compared to CRT group. However, it is also possible that VRT can induce higher tension and proportionate load in the range of motion, and consequently, greater myostatin and follistatin responses among some participants.

Considering the resting hormonal shifts to a more anabolic state, improvements in muscle mass and strength would be expected [40]. Accordingly, the findings of the present study indicated that there were significant correlations between FFM with anabolic and catabolic hormones and myokines including IGF-1, cortisol, myostatin, and follistatin (Fig. 5). Nevertheless, it was not the case for GH and this absence of association again stresses on the need for further identifying the mechanisms by which GH is regulated. We also found conjunct enhancements in muscular strength both in the bench press and squat. The VRT and CRT groups increased muscular strength (both bench press and squat) by about 30% and 25%, respectively (*p *= 0.001, Fig. 4). These findings demonstrate a comparable effect of chain-loaded VRT relative to CRT using only free weights, despite there being some evidence favoring the use of VRT (Fig. 4b, d).

These data extend on the findings of a previous study [13] that investigated the effects of 24 weeks of RT using a similar protocol to our study, among gender-mixed participants. The authors concluded that there was no significant difference between free weight and elastic-loaded RT in the magnitude of strength gains. Comparing to Bellar et al. [12], although unlike our study, they found significant difference between VRT and CRT groups, VRT-induced strength gains in bench press were quite similar to our study (9.95 vs. 9.41 kg). However, we noted greater gains for CRT group compared to those of them (8.50 vs. 7.56 kg).

One limitation of the present study is that the training cycle did not correspond to a certain phase of the menstrual cycle for each participant, but the length of the intervention (8 weeks) theoretically would have spanned two menstrual cycles in eumenorrheic women, meaning that the pre- and post-intervention testing likely occurred in similar menstrual phases for each participant. However, future research may wish to plan around the menstrual cycles of each individual participant to account for naturally occurring shifts in hormonal balance. Additionally, force output was not measured during training. Since the rationale of VRT lies in an increase, or at least the maintenance of, force throughout the entire range of motion, future studies should determine whether this occurs over time, as it is possible that neuromuscular adaptations may have occurred outside of 1-RM changes. Finally, although participants were asked to maintain their previous regular lifestyle physical activity and diet, it is impossible to know for sure whether they followed these instructions during the entire period of intervention. Therefore, similar to other studies on training programs, this could be considered as a limitation, but we do not believe that it has had major impacts on our findings.

## Conclusion

The findings of the present study support the hypothesis that incorporating chain-loaded VR into a periodized RT program can produce significant improvements in muscular strength. The present study expands upon this recommendation to suggest that chain-loaded VRT can be an effective alternative of CRT (using free weights) for untrained young women, as the findings demonstrate that VRT appears to induce comparable strength improvements to CRT among women with limited background in RT, even suggesting some advantages favoring VRT.

## Data Availability

The datasets used and analysed during the current study are available from the corresponding author on reasonable request.

## Abbreviations

1RM: One-repetition maximum
CI: Confidence interval
Con: Control
CRT: Constant resistance training
ES: Effect size
FFM: Fat free mass
GH: Growth hormone
IGF-1: Insulin-like growth factor 1
VRT: Variable resistance training

## References

[1] Kraemer WJ, Ratamess NA, Komi P (2003). Endocrine responses and adaptations to strength and power training. Strength and power in sport.

[2] Kraemer WJ, Ratamess NA (2005). Hormonal responses and adaptations to resistance exercise and training. Sports Med.

[3] Jensky NE, Sims JK, Dieli-Conwright CM, Sattler FR, Rice JC, Schroeder ET (2010). Exercise does not influence myostatin and follistatin mRNA expression in young women. J Strength Cond Res..

[4] Kraemer WJ, Marchitelli L, Gordon SE, Harman E, Dziados JE, Mello R (1990). Hormonal and growth factor responses to heavy resistance exercise protocols. J Appl Physiol.

[5] Chikani V, Ho K (2013). Action of GH on skeletal muscle function: molecular and metabolic mechanisms. J Mol Endocrinol.

[6] Walker S, Hulmi JJ, Wernbom M, Nyman K, Kraemer WJ, Ahtiainen JP (2013). Variable resistance training promotes greater fatigue resistance but not hypertrophy versus constant resistance training. Eur J Appl Physiol.

[7] Fleck SJ, Kraemer W. Designing resistance training programs, 4E: Human Kinetics; 2014.

[8] Berning JM, Coker CA, Adams KJ (2004). Using chains for strength and conditioning. Strength Cond J..

[9] Cronin J, McNAIR P, Marshall R (2003). The effects of bungy weight training on muscle function and functional performance. J Sports Sci.

[10] Andersen V, Fimland MS, Kolnes MK, Jensen S, Laume M, Saeterbakken AH (2016). Electromyographic comparison of squats using constant or variable resistance. J Strength Cond Res..

[11] Mina MA, Blazevich AJ, Giakas G, Kay AD (2014). Influence of variable resistance loading on subsequent free weight maximal back squat performance. J Strength Cond Res..

[12] Bellar DM, Muller MD, Barkley JE, Kim C-H, Ida K, Ryan EJ (2011). The effects of combined elastic-and free-weight tension vs free-weight tension on one-repetition maximum strength in the bench press. J Strength Cond Res..

[13] Shoepe T, Ramirez D, Rovetti R, Kohler D, Almstedt H (2011). The effects of 24 weeks of resistance training with simultaneous elastic and free weight loading on muscular performance of novice lifters. J Hum Kinet..

[14] Wallace BJ, Bergstrom HC, Butterfield TA (2018). Muscular bases and mechanisms of variable resistance training efficacy. Int J Sports Sci Coa..

[15] Soria-Gila MA, Chirosa IJ, Bautista IJ, Baena S, Chirosa LJ (2015). Effects of variable resistance training on maximal strength: a meta-analysis. J Strength Cond Res..

[16] Faul F, Erdfelder E, Lang A, Buchner A (2007). G*Power 3: a flexible statistical power analysis program for the social, behavioral, and biomedical sciences. Behav Res Methods.

[17] Marfell-Jones M, Olds T, Stewart A, Carter J (2006). International standards for anthropometric assessment (revised 2006).

[18] Jackson AS, Pollock ML, Ward A (1980). Generalized equations for predicting body density of women. Med Sci Sports Exerc.

[19] Siri WE (1956). The gross composition of the body Advances in biological and medical physics.

[20] Brzycki M (1993). Strength testing—predicting a one-rep max from reps-to-fatigue. J Health Phys Ed Rec Dance..

[21] Wallace BJ, Winchester JB, McGuigan MR (2006). Effects of elastic bands on force and power characteristics during the back squat exercise. J Strength Cond Res..

[22] Nakagawa S, Cuthill IC (2007). Effect size, confidence interval and statistical significance: a practical guide for biologists. Biol Rev.

[23] Hedges LV, Olkin I (2014). Statistical methods for meta-analysis.

[24] Cohen J. Statistical Power Analysis for the Behavioral Sciences, (L. Erlbaum Associates, Hillsdale, NJ). Erlbaum Associates Hillsdale, NJ.; 1988.

[25] Kraemer WJ, Fleck SJ, Dziados JE, Harman EA, Marchitelli LJ, Gordon S (1993). Changes in hormonal concentrations after different heavy-resistance exercise protocols in women. J Appl Physiol.

[26] Marx JO, Ratamess NA, Nindl BC, Gotshalk LA, Volek JS, Dohi K (2001). Low-volume circuit versus high-volume periodized resistance training in women. Med Sci Sports Exerc.

[27] Wideman L, Weltman JY, Hartman ML, Veldhuis JD, Weltman A (2002). Growth hormone release during acute and chronic aerobic and resistance exercise. Sports Med.

[28] Bouchard C, Rankinen T (2001). Individual differences in response to regular physical activity. Med Sci Sports Exerc.

[29] Jones N, Kiely J, Suraci B, Collins D, De Lorenzo D, Pickering C (2016). A genetic-based algorithm for personalized resistance training. Biol Sport..

[30] Marang CP (2018). The effects of elastic variable resistance training on chronic endocrine adaptations in resistance-trained men.

[31] Hayes LD, Bickerstaff GF, Baker JS (2010). Interactions of cortisol, testosterone, and resistance training: influence of circadian rhythms. Chronobiol Int.

[32] McMurray R, Eubank T, Hackney A (1995). Nocturnal hormonal responses to resistance exercise. Eur J Appl Physiol Occup Physiol..

[33] Roth SM, Martel GF, Ferrell RE, Metter EJ, Hurley BF, Rogers MA (2003). Myostatin gene expression is reduced in humans with heavy-resistance strength training: a brief communication. Exp Biol Med..

[34] Walker KS, Kambadur R, Sharma M, Smith HK (2004). Resistance training alters plasma myostatin but not IGF-1 in healthy men. Med Sci Sports Exerc.

[35] Costa A, Dalloul H, Hegyesi H, Apor P, Csende Z, Racz L (2007). Impact of repeated bouts of eccentric exercise on myogenic gene expression. Eur J Appl Physiol.

[36] Saremi A, Gharakhanloo R, Sharghi S, Gharaati M, Larijani B, Omidfar K (2010). Effects of oral creatine and resistance training on serum myostatin and GASP-1. Mol Cell Biol.

[37] Ma K, Mallidis C, Bhasin S, Mahabadi V, Artaza J, Gonzalez-Cadavid N (2003). Glucocorticoid-induced skeletal muscle atrophy is associated with upregulation of myostatin gene expression. Am J Physiol Endocrinol Metab.

[38] Lee S-J, McPherron AC (2001). Regulation of myostatin activity and muscle growth. Proc Natl Acad Sci.

[39] Attarzadeh Hosseini SR, Moeinnia N, Motahari Rad M (2017). The effect of two intensities resistance training on muscle growth regulatory myokines in sedentary young women. Obes Med..

[40] Mulligan SE, Fleck SJ, Gordon SE, Koziris LP, Triplett-McBride NT, Kraemer WJ (1996). Influence of resistance exercise volume on serum growth hormone and cortisol concentrations in women. J Strength Cond Res..

